# Progress in the Treatment of Alzheimer’s Disease Is Needed – Position Statement of European Alzheimer’s Disease Consortium (EADC) Investigators

**DOI:** 10.14283/jpad.2024.153

**Published:** 2024-08-11

**Authors:** Frank Jessen, M. G. Kramberger, D. Angioni, D. Aarsland, M. Balasa, K. Bennys, M. Boada, M. Boban, A. Chincarini, L. Exalto, A. Felbecker, K. Fliessbach, G. B. Frisoni, A. J. Garza-Martínez, T. Grimmer, B. Hanseeuw, J. Hort, A. Ivanoiu, S. Klöppel, L. Krajcovicova, B. McGuinness, P. Mecocci, A. de Mendonca, A. Nous, P.-J. Ousset, C. Paquet, R. Perneczky, O. Peters, M. Tabuas-Pereira, F. Piazza, D. Plantone, M. Riverol, A. Ruiz, G. Sacco, I. Santana, N. Scarmeas, E. Solje, E. Stefanova, S. Sutovsky, W. van der Flier, T. Welsh, A. Wimo, B. Winblad, L. Frölich, S. Engelborghs

**Affiliations:** 1 Department of Psychiatry, University of Cologne, Medical Faculty, Kerpener Strasse 62, 50937 Cologne, Germany; 2 German Center for Neurodegenerative Diseases (DZNE), Bonn/Cologne, Germany; 3 Department of Neurology, University Medical Centre Ljubljana, Ljubljana, Slovenia; 4 Faculty of Medicine, University of Ljubljana, Ljubljana, Slovenia; 5 Division of Clinical Geriatrics, Department of Neurobiology, Care Sciences and Society, Karolinska Institutet, Stockholm, Sweden; 6 Institut Hospitalo Universitaire HealthAge, Alzheimer’s Disease Research and Clinical Center, Toulouse University Hospital, Toulouse, France; 7 Center for Epidemiology and Research in Population Health (CERPOP), University of Toulouse, INSERM UMR1295, UPS, Toulouse, France; 8 Department of Psychological Medicine, Institute of Psychiatry, Psychology and Neuroscience, King’s College London, London, UK; 9 Department of Old Age Psychiatry, Centre for Age-Related Medicine, Stavanger University Hospital, Stavanger, Norway; 10 Alzheimer’s Disease and other Cognitive Disorders Unit, Neurology Service, Hospital Clínic, FRCB-IDIBAPS, University of Barcelona, Barcelona, Spain; 11 Resource and Research Memory Center (CMRR), Department of Neurology, Montpellier University Hospital, 80 avenue Augustin Fliche, Montpellier, France; 12 Ace Alzheimer Center Barcelona, Universitat Internacional de Catalunya, Barcelona, Spain; 13 Networking Research Center on Neurodegenerative Diseases (CIBERNED), Instituto de Salud Carlos III, Madrid, Spain; 14 University Department of Cognitive Neurology Referral Center for Cognitive Neurology Zagreb School of Medicine, Neurophysiology Croatian Ministry of Health University Hospital Centre, Zagreb, Croatia; 15 Istituto Nazionale di Fisica Nucleare (INFN), Genova, Genova, Italy; 16 Department of Neurology, University Medical Center Utrecht and Utrecht University, Utrecht, The Netherlands; 17 Department of Neurology, Kantonsspital St. Gallen, Gallen, Switzerland; 18 Department of Old Age Psychiatry and Cognitive Disorders, University of Bonn Medical Center, Bonn, Germany; 19 Memory Center, University of Geneva and University Hospitals, Geneva, Switzerland; 20 Servicio de Geriatría, Hospital Universitario Ramón y Cajal (IRYCIS), Madrid, Spain; 21 School of Medicine and Health, Klinikum rechts der Isar, Centre for Cognitive Disorders, Department of Psychiatry and Psychotherapy, Technical University of Munich, Munich, Germany; 22 Department of Neurology, Cliniques Universitaires Saint-Luc, Brussels, Belgium; 23 Institute of Neuroscience, UC Louvain, Brussels, Belgium; 24 Memory Clinic, Department of Neurology, Second Faculty of Medicine, Charles University and Motol University Hospital, Prague, Czech Republic; 25 INDRC, International Neurodegenerative Disorders Research Center, Prague, Czech Republic; 26 University Hospital of Old Age Psychiatry and Psychotherapy, University of Bern, Bern, Switzerland; 27 First Department of Neurology, Faculty of Medicine, St. Anne’s University Hospital, Masaryk University, Brno, Czech Republic; 28 Centre for Public Health, Queen’s University Belfast, Belfast, UK; 29 Institute of Gerontology and Geriatrics, Department of Medicine and Surgery, University of Perugia, Perugia, Italy; 30 Neurology Department, Faculty of Medicine, University of Lisbon, Lisbon, Portugal; 31 Department of Neurology, Universitair Ziekenhuis Brussel (UZ Brussel), Brussels, Belgium; 32 Center for Neurosciences, Vrije Universiteit Brussel, Brussels, Belgium; 33 Cognitive Neurology Centre, groupe hospitalier Saint-Louis-Lariboisière-Fernand-Widal, université de Paris, Paris, France; 34 Department of Psychiatry and Psychotherapy, University Hospital, LMU Munich, Munich, Germany; 35 German Center for Neurodegenerative Diseases (DZNE), Munich, Germany; 36 Aging Epidemiology (AGE) Research Unit, School of Public Health, Imperial College London, London, UK; 37 Sheffield Institute for Translational Neuroscience (SITraN), University of Sheffield, Sheffield, UK; 38 Department of Psychiatry and Psychotherapy, Charité - Universitätsmedizin Berlin, Berlin, Germany; 39 German Center for Neurodegenerative Diseases (DZNE), Berlin, Germany; 40 Department of Neurology, Dementia Clinic, Centro Hospitalar e Universitário de Coimbra, Coimbra, Portugal; 41 CAA and AD Translational Research and Biomarkers Lab, School of Medicine, University of Milano-Bicocca, Monza, Italy; 42 Department of Medicine, Surgery and Neuroscience, University of Siena, Siena, Italy; 43 Department of Neurology, Clínica Universidad de Navarra, Madrid, Spain; 44 Instituto de Investigación Sanitaria de Navarra (IdiSNA), Recinto del Hospital Universitario de Navarra, Pamplona, Spain; 45 Département d’Orthophonie, UFR Médecine de Nice, Nice, France; 46 CoBTeK, Université Côte d’Azur, Nice, France; 47 Service Clinique Gériatrique de Soins Ambulatoires, Centre Hospitalier Universitaire de Nice, Centre Mémoire Ressources et Recherche, Université Côte d’Azur, Nice, France; 48 Neurology Department, CHUC-Centro Hospitalar e Universitário de Coimbra, Coimbra, Portugal; 49 FMUC-Faculty of Medicine, University of Coimbra, Coimbra, Portugal; 50 CNC-Center for Neuroscience and Cell Biology, University of Coimbra, Coimbra, Portugal; 51 1st Department of Neurology, Aiginition Hospital, National and Kapodistrian University of Athens Medical School, Athens, Greece; 52 Taub Institute for Research in Alzheimer’s Disease and the Aging Brain, The Gertrude H. Sergievsky Center, Department of Neurology, Columbia University, New York, USA; 53 Institute of Clinical Medicine - Neurology, University of Eastern Finland, Kuopio, Finland; 54 Neuro Center – Neurology, Kuopio University Hospital, Kuopio, Finland; 55 Medical Faculty, Neurology clinic CCS, University of Belgrade, Belgrade, Serbia; 56 1st Department of Neurology, Faculty of Medicine, Comenius University and University Hospital, Bratislava, Slovakia; 57 Alzheimer Center Amsterdam, Neurology, Vrije Universiteit Amsterdam, Amsterdam UMC Location VUmc, Amsterdam, The Netherlands; 58 Amsterdam Neuroscience, Neurodegeneration, Amsterdam, The Netherlands; 59 Bristol Medical School (THS), University of Bristol, Bristol, UK; 60 RICE - The Research Institute for the Care of Older People, Bath, UK; 61 Royal United Hospitals Bath NHS Foundation Trust, Bath, UK; 62 Department of Neurobiology, Care Sciences and Society, Center for Alzheimer Research, Division of Neurogeriatrics, Karolinska Institutet, Solna, Sweden; 63 Centre for Research and Development, Uppsala University, Gävle, Sweden; 64 Department of Geriatric Psychiatry (LF), Central Institute of Mental Health, Medical Faculty Mannheim, University of Heidelberg, Mannheim, Germany; 65 Department of Biomedical Sciences, University of Antwerp, Antwerp, Belgium; 66 Department of Neurology, Universitair Ziekenhuis Brussel (UZ Brussel), Brussel, Belgium; 67 Neuroprotection and Neuromodulation (NEUR) Research Group, Center for Neurosciences (C4N), Vrije, Universiteit Brussel (VUB), Brussel, Belgium

**Keywords:** Alzheimer’s disease, mild cognitive impairment, disease modifying treatment, β-amyloid-targeting treatment, amyloid imaging related abnormalities

## Abstract

β-amyloid-targeting antibodies represent the first generation of effective causal treatment of Alzheimer’s disease (AD) and can be considered historical research milestones. Their effect sizes, side effects, implementation challenges and costs, however, have stimulated debates about their overall value. In this position statement academic clinicians of the European Alzheimer’s Disease Consortium (EADC) discuss the critical relevance of introducing these new treatments in clinical care now. Given the complexity of AD it is unlikely that molecular single-target treatments will achieve substantially larger effects than those seen with current β-amyloid-targeting antibodies. Larger effects will most likely only be achieved incrementally by continuous optimization of molecular approaches, patient selection and combinations therapies. To be successful in this regard, drug development must be informed by the use of innovative treatments in real world practice, because full understanding of all facets of novel treatments requires experience and data of real-world care beyond those of clinical trials. Regarding the antibodies under discussion we consider their effects meaningful and potential side effects manageable. We assume that the number of eventually treated patient will only be a fraction of all early AD patients due to narrow eligibility criteria and barriers of access. We strongly endorse the use of these new compound in clinical practice in selected patients with treatment documentation in registries. We understand this as a critical step in advancing the field of AD treatment, and in shaping the health care systems for the new area of molecular-targeted treatment of neurodegenerative diseases.

## The increasing societal burden of dementia

Of the 450 million people living in the European Union (EU), one in five is 65 years or older. One in three will be 65 or older in 2050 (https://ec.europa.eu/eurostat/). The current estimate of 7.8 million people living with dementia in the EU will almost double to 14.3 million in 2050 (www.alzheimer-europe.org). A recent meta-analysis of 17 European studies reported annual costs of dementia of up to 74,000 € per person in the severe disease stage. More than 90% of the costs are related to care, while less than 10% are related to diagnostic procedures and medical treatment (1).

## Dementia risk reduction and effects on dementia prevalence

A meta-analysis of epidemiological studies concluded that 40% of the population attributable risk of dementia is explained by potentially modifiable risk factors (2). Also, there is evidence that the age-specific prevalence and incidence rates of dementia are declining in industrialized countries (3). The magnitude of further reduction in age-specific incidence and prevalence of dementia, however, is uncertain, because some modifiable risk factors have already been successfully lowered at the population level over the past decades (e.g. smoking, hypertension) while others are on the rise (e.g. obesity, diabetes). Moreover, even under the assumption of a further decrease in age-specific incidence and prevalence of dementia, the disproportional demographic changes in Europe will override this effect and the burden of dementia for societies will continue to grow (https://ec.europa.eu/eurostat/). Approaches that slow the disease course and delay the time to moderate and severe dementia stages at an individual level are urgently needed.

## Causes of dementia and new treatments

Alzheimer’s disease (AD), the most common cause of dementia, is defined by deposition of extracellular β-amyloid as plaques and intraneuronal aggregation of phosphorylated tau protein with subsequent neurodegeneration (4). Cerebrospinal fluid (CSF) and brain imaging (positron emission tomography, PET) biomarkers are available to detect both pathologies even before dementia at the stage of mild cognitive impairment (MCI). Blood-based biomarkers are under development, which will increase the accessibility of a molecular AD diagnosis (5, 6).

Recently, it has been demonstrated for the first time that monoclonal antibodies against specific epitopes of aggregated β-amyloid reduce amyloid deposits in the brain and slow the progression of symptoms of the disease. Aducanumab was the first antibody to show the association of amyloid lowering with slowing of symptomatic decline in a phase 2 clinical trial (7). The phase 3 clinical trial program of aducanumab comprised two parallel studies (EMERGE, ENGAGE), which were terminated early after a futility analysis. In both trials, strong amyloid reduction was observed, but the clinical data were inconclusive (8). The United States Food and Drug Administration (FDA) granted accelerated approval based on the amyloid reduction only with the requirement for clinical proof of efficacy.

Lecanemab is the first antibody with demonstration of clinical efficacy in a phase 3 program (9). While it is fully approved by the FDA and by the regulatory bodies of Japan, China and South Korea, the Committee for Medical Products for Human Use (CHMP) of the European Medicines Agency (EMA) recommended against approval in July 2024. Donanemab, the second amyloid-targeting antibody with proof of efficacy has been fully approved by the FDA in July 2024. This is a historical milestone in AD research as it is the first time that a causative treatment shows slowing of symptom progression. The current antibodies can be considered the first generation of a new era of molecular-targeted treatment of AD and most likely of other neurodegenerative diseases given that 127 drugs targeting β-amyloid as well as several other molecular mechanisms, are currently in clinical development (10).

## Patient groups for β-amyloid-targeting antibodies

β-amyloid-targeting treatment aims at slowing the biological and symptomatic progression of AD. The target population of recent clinical trials were patients at the early symptomatic disease stage, either MCI or mild dementia. From the biological perspective, early treatment is plausible, because the spread and dynamics of pathology are less advanced at earlier stages, and more brain tissue is still preserved. Clinically, early treatment potentially maintains a stage of less impairment with more autonomy, higher independence, and lower costs of care. The efficacy of the new treatments in moderate and severe dementia is not studied and therefore remains unclear. It is likely, however, that the effects will be smaller than at earlier stages.

## Outcomes of the clinical trials

The 18-month phase 3 clinical trials of the current β-amyloid-targeting antibodies with evidence for efficacy used validated clinical instruments for measuring cognition and daily function as primary and secondary endpoints (9, 11). The Clinical Dementia Rating-Sum of Boxes (CDR-SOB) is most commonly used in early symptomatic AD trials and was the primary outcome of the lecanemab phase 3 clinical study (CLARITY-AD) (12). The phase 3 clinical trial of donanemab (TRAILBLAZER-ALZ 2) used the Integrated Alzheimer Disease Rating Scale (iADRS) as the primary endpoint (13). The CDR-SOB served as a key secondary endpoint. Both assessments (CDR-SOB, iARDS) are based on cognitive testing and interviews with patients and study partners by expert clinicians (raters). Additional instruments for assessing clinical symptoms were used as secondary endpoints in both trials. The primary biological readout of these studies was change a in β-amyloid plaques load measured by PET (9, 11).

## Effects and effect sizes

In CLARITY-AD and TRAILBLAZER-ALZ 2, there was consistent and significant slowing of decline on all clinical outcomes by antibody treatment compared with placebo. This was paralleled by a strong reduction of cerebral amyloid load to a level of what is considered amyloid-negative in most participants (9, 11).

The effect was a reduction in decline in comparison to placebo at the end of the studies of 27% on the CDR-SOB for lecanemab and of 35% on the iADRS for donanemab in the primary analysis group of TRAILBLAZER-ALZ 2, which were patients with low to medium tau deposition on PET. The absolute difference in favor of the drug vs. placebo in the CDR-SOB was 0.45 points for lecanemab and 0.68 points for donanemab in the low to medium tau group (0.67 in the full sample) (9, 11).

## Clinical meaningfulness of the effects

There is an ongoing discussion about the clinical meaningfulness of the magnitude of the observed effects. The following points are important to consider: the CDR-SOB, the iADRS and all other scales used as clinical outcomes measure inherently meaningful features of the disease, namely impairment in cognition and function, which are the core symptoms that define the clinical manifestation of AD.

Regarding the most used CDR-SOB, studies have linked the magnitude of change on this scale to clinically relevant changes on external references (anchors). Based on the US National Alzheimer’s Coordinating Centers (NACC) database, one study reported a mean change in the CDR-SOB score of 0.98 in MCI-AD patients and of 1.63 in AD patients with mild dementia, who showed a minimal clinically important decline over 12 months according to physician judgement (14). A second study based on the Donepezil/Vitamin E in MCI clinical trial data (ADC-008 NCT00000173) calculated a meaningful change of the CDR-SOB of 0.64 points over 12 months by anchoring it to the MCI-Clinical Global Impression of Change Scale (MCI-CGIC) and of 1.08 by anchoring it to the Global Deterioration Scale (GDS) (15). These results show that the estimates differ substantially between MCI and mild dementia, indicating lower sensitivity to change in MCI, and that the estimates depend on the sample, the context of data acquisition and the selected anchor. It also needs to be recognized that the external anchors in these studies are clinician-based categorical judgements (i.e. worse, not worse) with imperfect reliability and validity. As such, these estimates can only serve as rough guidance. As a side note, in these studies, clinical meaningful change was defined by the treating physician or rater, not by the patient or the care partner.

According to these estimates, the placebo group in CLARITY-AD showed a clinically relevant decline (1.66 points), while the lecanemab group showed a borderline decline (1.21 points) (9). The placebo group in TRAILBLAZER-ALZ 2 showed a clearly relevant decline (1.82 points), while the donanemab group showed a decline of only borderline clinical relevance (1.16 points) (11). The higher number of patients with a CDR global score of 0.5 (indicating milder impairment in the range of MCI) in CLARITY-AD (80.8%) compared with TRAILBLAZER-ALZ 2 (65.7%) may explain the smaller effect of lecanemab given the lower sensitivity of change of the CDR-SOB in MCI compared with mild dementia.

A different way to interpret the effect size is a time-to-event analysis, which is common in other medical fields, such as oncology. The hazard ratios (HR) of progressing to a more severe clinical stage (MCI to mild dementia, mild dementia to moderate dementia based on CDR ratings) within 18 months were 0.69 for lecanemab (risk reduction of 31%) and 0.61 for donanemab in the low to medium tau group (risk reduction of 39%) (full sample: 0.63) (9, 11). From the societal point of view, this risk reduction is important given the increase of care-related costs with advancing disease stages. For comparison with other fields of medicine, a recent meta-analysis across 92 FDA-approved anti-cancer drugs reported a HR for progression-free survival of 0.52 (16).

Complementary approaches of assessing meaningfulness are patient-reported outcome measures (PROM). In CLARITY-AD, self- and care partner-reported quality of life and caregiver burden were assessed. Significant superiority of lecanemab was observed in all three domains (17).

## Expected effect sizes of new treatments

AD is a highly complex disease with several relevant molecular pathways beyond β-amyloid and tau aggregation. A recent genome-wide association study (GWAS) identified 75 significant risk loci. A pathway analysis revealed 33 significant gene sets and identified a strong role of the innate immune system and microglia-dependent endocytosis (18). In addition, AD pathology is driven by environmental and lifestyle risk factors as well as general mechanisms of aging. Consequently, current drug development has extended to inflammation, metabolic regulation, oxidative stress, synaptic protection, neurotransmitter imbalance and proteostasis complementary to β-amyloid aggregation and tau pathology (10). Given the multifaceted causes underlying AD and the fact that at the time of symptom manifestation, the disease pathology has already progressed for several years, it is not very likely that targeting a single molecular mechanism at the stage of early symptomatic AD will have much stronger effects than what is now observed in β-amyloid-targeting antibody trials. Larger effects will most likely only be achieved by combination therapies, individualized biomarker-guided patient selection, and by long-term treatment from the very early disease stage onwards. This assumption clearly suggests that the progress in treatments will be incremental and, given the long duration of trials needed in AD will extend over many years.

## Side effects of β-amyloid-targeting antibodies

The most discussed side effects of the current β-amyloid-targeting treatments are amyloid-related imaging abnormalities (ARIA), which are changes observed on magnetic resonance imaging (MRI). They are classified as ARIA-E and ARIA-H. The first refers to hyperintense signal abnormalities with evidence of brain swelling on T2-weighted MRI. ARIA-E is considered to reflect vasogenic edema due to antibody effects on amyloid in vascular walls often accompanying AD pathology (19). ARIA-H describes microbleeds or superficial siderosis also considered to be related to cerebral amyloid angiopathy (CAA) and CAA-related inflammation (CAA-ri) (20, 21). In the trials, the occurrence of ARIA led to discontinuation and re-start of dosing following specific protocols.

A recent summary of ARIA related to lecanemab treatment in CLARITY AD including the open label extension study reported an ARIA-E frequency of up to 34.5% in APOE4 homozygous with 14.2% being recurrent (6.5% and 0.8% in APOE4 non-carriers). 92% occurred in the first six months of treatment, 81% resolved within four months. The frequency of symptomatic ARIA-E was 3.3% (11.2% in APOE4 homozygous) with mild to moderate and transient symptoms including headache, confusion, dizziness, vision changes, nausea, aphasia and weakness. Individual cases of seizure were reported in association with ARIA, however, the frequency of seizures in lecanemab-treated patients was the same as in the placebo group (0.6%) (22). In APOE4 homozygous the frequency of ARIA-H was 39.8% in lecanemab-treated group and in 21.1% in the placebo group (11.9% and 3.8% in APOE4-non-carriers). Of all lecanemab-treated patients, 1.7% showed symptomatic ARIA-H. Intracerebral hemorrhage occurred in 0.5% (8 of 1612) of lecanemab-treated cases and in 0.1% (1 of 897) of the placebo group, of which two in the lecanemab group and one in the placebo had fatal outcomes. Of the two fatal cases under lecanemab, one received tissue plasminogen activator (tPA) treatment before the hemorrhage and one received anticoagulant therapy (22).

In TRAILBLAZER-ALZ 2, ARIA-E occurred in 40.6% of APOE4 homozygous (15.7% in non-carriers). 6.1% of all donanemab-treated patients had symptomatic ARIA-E (25.4% of all ARIA-E cases). First events of ARIA-E resolved in 98% of the cases after a mean of 72.4 days. ARIA-H was observed in 36.4% of all treated patients and 13.6% in the placebo group. Intracranial hemorrhage occurred in 0.4% of treated cases (3 of 853) in 0.2% in the placebo group (2 of 874). Three participants in the donanemab group died in relation to severe ARIA-E (n=1), severe ARIA-E and ARIA-H (n=1) and severe ARIA-E and ARIA-H with intracranial hemorrhage (n=1). Two were APOE4 heterozygous carriers and one was an APOE4 non-carrier. None had received anticoagulant or anti-platelet medications (11).

These data show that ARIA-E and ARIA-H are common, particularly in APOE4 carriers. They also show that transient clinical symptoms of mild to moderate severity occur in a fraction of all ARIA cases. Severe clinical side effects even with fatal outcome occur in very rare cases. Every effort must be taken to understand the underlying mechanisms of these severe events. Risk factors for ARIA must be known to prescribing physicians, and biomarker, which indicate ARIA-risk are highly desirable (22). However, the trials also demonstrate that with careful monitoring ARIA are manageable and patient can generally be treated safely. In our view, the perception that β-amyloid-targeting treatment with antibodies puts patient at high risk for dangerous side effect is not supported by data and misleading.

## Eligibility for treatment

In the United States (US) appropriate use recommendations (AUR) for aducanumab and lecanemab have been published to guide the clinical use of these antibodies with special consideration of patient selection and safety monitoring (23, 24). In addition to the proof of AD pathology by biomarkers in mildly symptomatic patients, the AURs list several eligibility criteria, which are oriented along the inclusion and exclusion criteria as well as safety findings of the clinical trials (9, 10). Key aspects are the level of impairment as defined by cognitive testing, co-medication, and eligibility for safety monitoring (i.e. lack of MRI contraindications).

A number of reports have estimated the proportion of individuals who would qualify for treatment. In the Mayo Study of Aging, 5.1%–17% of 237 patients with MCI or mild dementia and evidence for Aβ pathology were found to be eligible depending on the compound and the neuropsychological criteria used (25). In an analysis of the Karolinska Memory Clinic sample 27% of MCI cases and 28% of dementia cases would qualify for aducanumab treatment without consideration of MR-based exclusion criteria (26). An analysis of the database of the Centre for Dementia and Cognitive Decline (CDCD) in Brecia revealed 32.6% of with MCI patients to be potentially eligible for aducanumab treatment (27). Based on National Health Service data, it was estimated that 30.200 patients (3.1% of all dementia cases) would qualify for antibody treatment in the UK (28). These estimates show that among those patients at the early symptomatic stage of AD, only a fraction is eligible for the currently available β-amyloid-targeting antibodies.

## Access barriers

The identification of early-stage AD patients potentially eligible for amyloid-targeting treatment requires vigilance from patients, family members and physicians for early symptoms. While there is a general openness in primary care for early diagnosis, many barriers exist for early case finding. Frequent topics hindering early diagnosis at the non-specialized level include lack of time and knowledge about early AD as well as insufficient reimbursement (29). There is a substantial need to develop tools which allow correct detection of MCI and mild dementia in the non-specialized settings and to create an environment, in which the primary care physician is supported in the diagnostic process, counseling and care for patients with early-stage AD. A second barrier to early diagnosis of AD is access to an expert diagnosis including evaluation of CSF or PET biomarkers. The necessary capacities for diagnostics currently exceed what is available in almost all European countries (30). Plasma-based biomarkers may help to overcome challenges of diagnostic capacity in the future, but expert centers guiding the diagnostic process will still be required and present a bottleneck. With regard to treatment, infusion center capacity is a limiting factor. An additional challenge is the requirement of MRI side effect monitoring with five and - in case of ARIA - potentially even morse scans within the first year of treatment and additional scans subsequently, which is needed to guide dosing decisions including even cessation of treatment. As such, access to repeated MRI represents another bottleneck, which needs to be overcome in order to make effective treatment available.

Besides eligibility criteria and structural barriers of access other aspects will most likely impact on the number of patients eventually receiving treatment. Of relevance will be for instance the risk/benefit/effort/cost considerations of individual patients and care partners after the informed-consent process, lack of knowledge about new treatments on the level of patients and physicians, skepticism of consulting physician including recommendation against treatment, long distance or complicated traveling to facilities, out-of-the-pocket expenses in some healthcare systems and many other. All of these aspects most likely contribute to the lower than expected number of treated patients currently observed in the US, where antibodies are approved.

## Costs related to treatment

There has been a concern that the costs of diagnostics, treatment and monitoring per patient with the current β-amyloid-targeting antibodies will exceed the capacity of national health care systems. In a previous EADC paper, we concluded that the economic consequences would be considerable, if one third of the early AD-population would get access to treatment (31). Given the consideration above, however, all cost models carry uncertainties, because the real number of individuals who will be treated is difficult to estimate before the compounds enter healthcare.

In addition, prospective developments of treatments will potentially lower the costs. For example, subcutaneous application of antibodies, which is under development, will make infusion centers dispensable; treatment only until amyloid negativity, which was reached in 80% of all patients in the TRAILBLAZER-ALZ 2 trial after 18 months, would limit treatment duration per patient (for this, meaningful blood biomarkers for treatment-related amyloid lowering are needed); improvement in anti-amyloid treatment with regard to ARIA risk may make MRI-monitoring in the future unnecessary.

## Conclusion and outlook

With the advent of β-amyloid targeting treatments as the first generation of causal treatment of AD and the rapidly evolving biomarkers, the field of neurodegenerative diseases is entering a new era, which for the first time holds promise for effectively slowing the biological disease processes and eventually preventing severe clinical disease stages which are associated with high burden and costs of care. The complex biological nature of AD and other neurodegenerative diseases implies that the clinical effects of single-target treatments starting at the symptomatic stage of the disease will most likely be limited. We expect upcoming mono-targeted non-amyloid treatments to achieve effect sizes comparable to those of lecanemab and donanemab. It is likely that significantly stronger effects will only be achieved by combination therapies, by individualized biomarker-guided patient selection or by much earlier treatments. All these approaches require trial designs, which are more complex than the recent phase 3 clinical trials. Their development will need to build on current clinical trial evidence, but also capitalize on experience and knowledge gained by clinical use. Overall, the progress towards larger effect sizes will most likely be incremental and extend over several years. It is of critical importance for the research community as well as regulators, payers and the public to acknowledge these expectations. To eventually achieve the best outcome, we need strong and sustained investments in research and in the development of health care systems with the integration of innovations. We need to establish technologies and pathways of care that allow timely identification of patients who are eligible for and may benefit from these treatment and guarantee access to safe administration and monitoring. As in other fields, we must make use of real-world data collected in large-scale registries to better understand effects and risk of the upcoming treatments. With sustained commitment of all stakeholders, the field of neurodegenerative diseases will eventually evolve from symptomatic dementia care to early disease detection with effective causal treatment and delay or even prevention of late stage disease leading to reduced care dependency and cost savings for the society.
